# Using the gravitational mixed models to analyze the impact of China's foreign direct investment along with The Belt and Road countries on trade flows

**DOI:** 10.3389/fpsyg.2022.960722

**Published:** 2022-08-16

**Authors:** Te-Hsin Hsieh, Ye-Bin Zhu, Kuo-Lung Huang

**Affiliations:** ^1^School of International Business, Xiamen University Tan Kah Kee College, Xiamen, China; ^2^Institute of Human Resource Management, National Sun Yat-sen University, Kaohsiung, Taiwan

**Keywords:** The Belt and Road, panel data, foreign direct investment, trade flows, gravitational models

## Abstract

Since the “The Belt and Road” initiative was put forward in 2013, China's foreign investment growth rate has been greatly accelerated. In The Belt and Road context, many scholars used models to analyze the relationship between foreign direct investment, trade flows, and import and export trade. From literature reviews, it is found that previous scholars do not conform to reality and cannot be studied dynamically. Therefore, this study used the panel data of China's foreign direct investment and import and export trade in 40 countries along “The Belt and Road” from 2010 to 2019 to focuses on the impact of foreign direct investment (FDI) on trade flows, import trade and export trade. Regression analysis was carried out with the trade gravity model and Tinbergen's trade gravity model. In addition to model variables and arguments, the following control variables were adopted: exchange rate, natural resource rents, labor force population, differences in consumer ability, trade openness, and trade agreement signing. The results found that: (1) Foreign direct investment has a positive correlation with trade flow and import and export trade. (2) The labor force population has a negative correlation with trade flow, and import and export trade. (3) The expansion of China's economic scale can increase the scale of China's import and export trade, at the same time, the gap in consumption levels between the two countries will restrict the degree of import and export trade. (4) The possession of natural resources can also promote the development of trade.

## Introduction

In 2013, the national high-level cooperation initiative (“The Belt and Road”, B&R) was formally proposed, and the “Vision and Actions on Jointly Building Silk Road Economic Belt and twenty-first-Century Maritime Silk Road” was announced in 2015. At present, it runs through China's Eurasian continent, the Middle East, and Africa. In recent years, the B&R cooperation has entered a stable period, and investment and trade opportunities are emerging one after another. As of May 2020, China had signed Belt and Road cooperation documents with 138 countries and 30 international organizations. In 2019, Chinese domestic enterprises set up 11,000 overseas enterprises in countries along the B&R route, involving 18 major sectors of the national economy. In the same year, they achieved a direct investment of 18.69 billion, an increase of 8.63% over 2018. It accounted for 13.7% of the total foreign investment. With the deepening of trade, the single manufacturing and wholesale industry in the investment field has expanded to emerging industries such as finance, mining, and electric power, and the proportion has continued to increase. As of 2019, China's exports to partner countries were US$762.29 billion and imports were US$581.73 billion, with an average annual growth rate of 4.4%. According to a report released by the World Bank, the B&R will lift 7.6 million people out of extreme poverty and 32 million people out of moderate poverty in relevant countries, increasing the trade of participating countries by 2.8–9.7%, global trade by 1.7–6.2% and global income by 0.7–2.9%. With the continuous development of the B&R investment and trade, the development of foreign investment to multilateral import and export trade volume has been the main topic of discussion in the field of economics since the twenty-first century. The economic policy of opening up to the outside world is conducive to promoting the outlook of the world economy and bringing huge capital product opportunities to large, medium, and small import and export enterprises in China. Collaborative resources, foreign investment, and trade are an important part of the B&R.

In theory, import and export trade is interconnected, but most scholars' research focuses on the impact of investment on trade flows. Therefore, this study will analyze the impact of trade and investment on import and export trade based on many scholars' research on “export”. On the other hand, the effect of the relationship between the two will be different when the research is conducted under different models. This study uses Tinbergen's trade gravity model for analysis and comparison, which expands innovation and practical contributions. In practice, for one thing, The B&R research is beneficial to analyze and comment on the achievements under the new socialist development pattern with Chinese characteristics in the new era as a whole and provide reference directions for companies and society. For another, the results of the analysis provide a factor analysis on the influence of enterprises and society in foreign direct investment (FDI).

## Literature review

### Gravity model of trade

Scholars in the past used the gravity model of Trade (Baniya et al., 2020; Li et al., 2020; Shahriar et al., 2020) as well as the stochastic frontier gravity model (Li, 2016; Xu, 2017; Fang, 2018; Yang, 2020). They conducted researches such as China's import, export, trade and investment benefits for different countries along the B&R. Therefore, this study is based on the Gravitational model and uses Tinbergen's trade gravity model for analysis.

### Tinbergen's trade gravity model

The Trade gravity model is a method to analyze trade flow. It originated from Newton's famous theory of gravity. This study adopts the trade gravity model. Tinbergen's trade gravity model is a method of analyzing trade flows and is the most widely used model in international trade analysis. The basic formula comes from Newton's famous theory of gravitation.


$$TF_{ij}\;=\;(GDP_{i}*GDP_{j})/\text{disp}$$

Note: TF represents trade flow, *i* and *j* represent two countries respectively, and disp represents the distance between the two countries. The reasons for using the trade gravity model in this study are as the followings.

The use of panel data in transacting models is flexible and convenient, and is widely used in trade potential prediction, group effect analysis, pattern judgment and other fields (Guo, 2011), and the trade model is more explanatory. Gu (2001) believes that the gravity model can explain the phenomenon that traditional trade cannot explain. Therefore, the model provides a theoretical basis. On this basis, Tinbergen's trade gravity model, a Nobel Prize winner in 1969, is extended and transformed into a new gravity model, which can be used for various import and export trades. The Tinbergen trade model is an extension of the physical basis, and it mainly studied the impact of import and export trade. This study adopts this model to study the total import and export trade. The following is the formula.


$$X_{ij}\;=\;\kappa (GDP_{i}^{\alpha }*GDP_{j}^{\beta }/{\left(1+\text{edisp}\right)}^{\mu })$$

Note: *X* represents export, *I* and *j* represent two countries respectively, and disp represents the distance between the two countries.

### Regional industrial trade impact

Foreign scholars have also analyzed the impact of the B&R Initiative on various regions. Quoc et al. (2019) found that impact of China's global trade facilitation on Vietnam from the B&R Initiative. Shi and Cai (2020) studies the relationship between oil imports and investment, estimating that FDI can increase host countries' oil imports and diversify China's import sources. Ma et al. (2019) analysis found that China's direct investment in Europe boosted exports.

### The impact of institutional distance on trade

Institutional distance refers to the extent to which two countries differ in institutional rules, norms, and perceptions. It can be obtained by calculating the average of the six indicators in the global governance database and establishing a theoretical model. Lu (2021) used institutional constraints to analyze the current situation of investment and trade between China and the 27 EU member states from the perspectives of scale, country, and industrial structure. Xie (2021) analyzed data on foreign direct investment, import and export trade, and institutional distance from 42 countries. Liu (2020) constructed a fixed effect model from the perspective of institutional distance and analyzed the impact of export trade and the impact of import trade. Gong and Wang (2020) analyzed the impact of institutional distance on the export effect. The above studies illustrated the impact of investment on trade.

### The Belt and Road direct investment analysis of some areas

In the context of macro overall analysis, many scholars had made detailed analyses on individual countries along the B&R. The vertical analysis also showed the actual impact of foreign direct investment on countries along the route. Sun (2019) analyzed the impact of China's foreign direct investment on Cambodia's trade. Zhang (2020) studied China's foreign direct investment in Malaysia and found that direct investment has an impact on the creative effect of trade scale. Huang (2020) made an in-depth discussion on China Laos foreign direct Investment, which promoted the optimization and upgrading of trade structure.

Saeed et al. (2021) explored the potential effects of the B&R policy on trade flows in ASEAN countries and China.

### Research on the control variables for the relationship between the two

The effects of direct investment and trade-scale flows are complex. Many scholars studied factors such as investment motivation, income and technology. Xu (2019) found the relationship between overall investment and trade effects, and different income countries have different effects on different trade structures. Wang (2019) Foreign Direct Investment Analysis of the Impact of Foreign Direct Investment. Wu (2019) conducted a trade gravity model analysis of direct investment in 56 countries, and obtained the trade effect of direct investment. Tang (2021) analyzed the mechanism of direct investment export scale and export structure. Li (2021) found the relationship between investment and exports in model testing.

## Method and results

This study focuses on Empirical Research from China. Based on the data of China's foreign investment from 2010 to 2019, trade flows with 40 countries along the B&R, the Stata 16.0 economic data analysis tool is used to analyze the trade flow in panel data. Based on the analysis of the Gravity Model of Trade, Tinbergen's Trade Gravity Model are used to analyze the respective trade of import and export, to obtain the impact of investment on trade flow and the overall impact on import and export.

### Current situation analysis

#### Analysis of the current situation of direct investment and import and export

The period of this study is from 2010 to 2019, and the data of 40 countries along the B&R route are relatively complete. The B&R initiative was proposed in the study is 2013. The trade comparison before and after the proposal could better reflect the significant difference in the growth of investment or import and export.

##### Current status of direct investment

From 2010 to 2019, China's foreign direct investment showed a high level, a wide field, and a wide range. It promoted the rapid growth of Chinese enterprises by deepening the B&R. Therefore, this study analyzes China's investment stock and flow according to time, place, and investment scale.

##### Investment over the years

China's foreign direct investment shows a rapid growth trend along the B&R. China began to increase investment flow and investment stock. From US 30.4 billion dollars in 2013 to nearly US $200 million in 2017, it reached a peak, then increased by 12%, and finally became balanced. Based on the impact of the B&R initiative on the world economy, the gap between world economic growth and China's direct investment and China GDP growth were analyzed. Find that China's economic growth trend is almost parallel to the world trend. From 2018 to 2019, while reducing economic growth, the proportion of investment along the route also decreased from 10% to 9% (Data source: “Belt and Road” development cooperation report).

##### Classification of investment areas

China's foreign direct investment stock mainly comes from countries near-by. In 2018 and 2019, the proportion of China's investment in East Asia will reach 82% and 70% respectively. In 2019, the trade flows, investment flows mainly to Southeast Asian countries, and Japan's flows and stocks rank first, with US $181 million and US $220 billion respectively. They account for 25.2% and 43.3% of China's investment stock and flow respectively (Data source: Mark Database).

##### Investment industry summary

From 2013 to 2019, investment was more diversified, from a single field to multiple fields. The whole investment, manufacturing, wholesale and retail, construction and financial technology industries rank among the top five in the investment industry. They invested 6.79, 2.51, 2.24, 1.59, and 1.35 billion, respectively. Among them, the manufacturing industry accounted for 36.3% in the same year, with a year-on-year increase of 15.5%. Wholesale and retail trade accounted for 25.1%, an increase of 13.4% in the same year (Data source: 2019 Statistical Bulletin of China's Outward Direct Investment).

##### Current status of trade flow

Trade flows refer to total trade imports and trade exports. With the continuous updating and development of global economic policies, economic and trade behavior has attracted the attention of all countries after the B&R strategy was put forward. In 2019, Japan, South Korea, Vietnam, Malaysia, and Russia ranked in the top five in terms of total import and export trade with China. Next, the analysis will be done at the time level and the spatial distribution. Compared with the subsequent import and export trade, it has a general leading role.

##### Flow over the years

From 2016 to 2017, China's trade volume with the countries mentioned in this research has achieved a relatively breakthrough. After experiencing negative growth from 2015 to 2016, the growth of China's trade scale has accelerated, forming a staged improvement (Data source: UN trade).

##### Flow spatial distribution

Comparative analysis of 2010-2013 and 2013-2019. Before the signing of the trade agreement, China's transactions with central and Eastern Europe accounted for the vast majority. After the launch of the initiative in 2013, the proportion of trade flows with South Asia increased significantly from 2014 to 2015, and the proportion of trade flows with Southeast Asia increased from 2016 to 2017. Two years accounted for 48% and 50% respectively. By 2018, the proportion of East Asia will increase significantly. As of 2019, China's trade with East Asian countries has been relatively frequent. Among China's foreign exchanges and cooperation, Japan's trade with China ranked first, accounting for 2.37% of the total trade, then followed by South Korea (2.15%), Vietnam (1.2%), and Malaysia (0.9%) (Data source: UN trade).

##### Analysis of China's import status

From 2010 to 2019, China's imports (exports by country) to countries along the B&R achieved leapfrog growth. In terms of time scale, with the deepening of trade, from the signing of the initiative in 2013 to the growth in 2019, the import performance increased by 20.8% and remained stable in 2019. There is still a serious regional trade imbalance in China's imports to countries along the route. Before 2013, the import situation was diversified. From 2013 to 2019, the proportion of China's imports from South Asia continued to increase, from 17.6 to 20.9%. From 2013 to 2019, the proportion of import trade to Central Asia also increased from 13.2 to 21.2%. South Korea, Japan, Malaysia, Vietnam, and Russia ranked in the top five in terms of import trade volume to China in 2019. South Korea's import trade with China accounted for 20% of the country's total exports and is one of the main trading countries to China's import trade (Data source: UN trade).

##### Analysis of China's export status

In terms of time series, China's foreign trade continued to grow from 2013 to 2019. In the face of the volatile political situation and world economic recession, the growth of export trade gradually slowed down and increased, and finally remained unchanged (source: UN trade).

From 2010 to 2013, the main trade export object was South Asia, rising from 7 to 12%, an increase of 70%. From the signing of the initiative in 2013–2019, the proportion of South Asia has decreased significantly. By 2019, Central Asia has become the largest export sector in China, accounting for 29.4% of the five sectors (Data source: UN trade).

From the perspective of trading countries, the top five countries in China's foreign export trade volume in 2019 were Japan (1.43 billion), South Korea (1.1 billion), Vietnam (980 million), Singapore (540 million), and Malaysia (520 million), accounting for 70.9% of the total export volume. Most countries are China's neighbors, and some countries are in West Asia, which can better reflect China's B&R initiative (Data source: UN trade).

##### Impact factors of direct investment and trade relations

Based on the above status and analysis of the B&R direct investment from 2010 to 2019, this study incorporates relevant influencing factors into the model. Including model itself variables, import and export variables, and 5 domestic and foreign factors.

##### Degree of trade openness

The degree of trade openness refers to the degree of a country's economic and trade opening to the outside world. The degree of opening to the outside world (lnq) starts from the commodity market, that is, the import and export of foreign trade are relatively stable. Therefore, it is generally selected as the evaluation and measurement index of openness in the world. This study adopts the nominal degree of opening to the outside world, and its formula is the ratio of the country's total trade volume to the total GDP, that is, the country's total trade volume is directly proportional to the degree of opening to the outside world. Therefore, the degree of openness can indirectly reflect the impact of countries on trade after passing the project. Wang (2017) found that trade openness has a positive impact on it. Xin et al. (2019) showed that trade opening can expand the economic level and expand trade.

##### Trade agreement signed

From the past research literature, there is a correlation between signed trade agreements and trade exchanges. Therefore, this study looks for whether the countries along the route have signed an agreement with China from the free trade agreement(fta), and makes an empirical analysis. The source of it is the China free trade area service network. Wu (2018) found that the bilateral trade creation effect has strong characteristics, but the trade diversion effect is not strong.

##### Exchange rate

The exchange rate(lnnrr) here is the US dollar exchange rate collected by untraded. On the one hand, the change of income is caused by the change of exchange rate, which affects import and export trade, as well as the balance of payments and investment, and the resource of it is the World Bank. Li (2020) analyzed the exchange rate chain and direct investment of countries along the route. Lin (2016) found that the RMB exchange rate will not significantly increase the trade flow and expand the trade scale after studying the influencing factors and potential of the B&R countries.

##### Differences in consumer capabilities

The difference in consumption power (lndpcl) is calculated by subtracting the average GDP of each country and China's GDP in that year. Per capita GDP reflects the improvement of a country's economic growth quality and residents' income level, and directly affects the scale and level of international trade. Tang (2021) used the consumer ability difference index and found that it had a significant impact on the export effect.

##### Labor force population

The labor force(lnlab), from the World Bank, can consume through the income obtained to bring more GDP benefits and drive the import and export trade. Shi (2018) studied that different labor structure has a significant impact on China's service trade export. The aging labor structure is unfavorable to the development strategy of export trade. The development of trade among countries will also change with the change of labor force. Whether the labor force population will affect the development of trade flows is worth exploring. Therefore, this study uses the variable division of labor and population to convert and test the per capita GDP, and also uses it in empirical research.

##### Natural resource rents as a share of GDP

The amount of natural resources(lnnrr) in a country is inversely proportional to technological progress. In the past, scholars used the ratio of natural resource rent to GDP to describe the natural resource endowment of each country, it can be get from the World Bank. Wu (2019) added this variable to direct investment and studied the trade effect at the same time.

### An empirical research on the relationship between direct investment and trade flows

#### Data sources and variable descriptions

The sample data of this study are from the world trade organization, the World Bank, and CSP II databases. Due to the political and other factors of some countries, the countries that cannot fill in the data are deleted. The others listed as the B&R foreign direct investment (FDI) are used to analyze the trade flow and the data analysis of the trade influencing factors (fta,lnlab,lndpcl, lner,lnq,lnnrr). The data analysis period started from 2010 to 2019. Stata 16.0 economic data analysis tool is used to analyze the trade flow. For the import and export trade situation, Tinbergen's trade gravity model is used, and the formula is as the followings:


$$\begin{array}{l} lntf_{ic}\;=\;\beta_{1}lnGDP_{i}\;+\;\beta_{2}lnc\;+\;\beta_{3}lnofdi\;+\;\beta_{4}lndisp\;+\;\beta_{5}lnnrr\\ \;+\;\beta_{6}lnlab\;+\;\beta_{7}lnql\;+\;\beta_{8}lndpcl\;+\;\beta_{9}fta\;+\;\beta_{10}lnex\\ \;+\;\mu \;+\;v\;+\;\varepsilon \end{array}$$

lntf is the trade flow, which is the sum of imports and exports GDP_i_ (unit:10 billion) is the GDP value of each country in the current year, c is the GDP value of China in the current year (unit:10 billion), both of the results come from the World Bank. The lndpcl (unit: USD 100) is the distance between each country and the capital of China, and lnofdi is the foreign direct investment (unit: billion US dollars), the source of it is the statistical bulletin of China's foreign direct investment. The lnlab represents the number of workers in the country, and fta is a dummy variable, indicating whether the country has signed a trade agreement with China. The lndisp as well as the ln1edisp ln(1+edisp) stand for the distance between the capital and China (unit: thousands of meters), where can be obtained by CSPII. The lner is the exchange rate (unit: dollar) and the lnnrr is the natural resource (unit:%). μrepresents the individual fixed effect, the lnq is the degree of trade openness, the v represents the time fixed effect and ε is the error term. For the import and export trade situation, Tinbergen's trade gravity model is used, and the formula is as follows:


$$\begin{array}{l} Inm\;=\beta_{1}lnGDP_{i}\;+\;\beta_{2}lnc\;+\;\beta_{3}ln\left(1\;+\;edisp\;\right)\;+\;\beta_{4}lnofdi\\ \;+\;\beta_{5}lner+\;\beta_{6}lnlab\;+\;\beta_{7}fta\;+\;\beta_{8}lnq\;+\;\beta_{9}lner\;+\;\beta_{10}lnq\\ \;+\;\mu \;+\;v\;+\;\varepsilon \; \end{array}$$

$$\begin{array}{l} Inx\;=\;\beta_{1}lnGDP_{i}\;+\;\beta_{2}lnc\;+\;\beta_{3}ln\left(1\;+\;edisp\;\right)\;+\;\beta_{4}lnofdi\\ \;+\;\beta_{5}lnlab\;+\;\beta_{6}lndpcl\;+\;\beta_{7}fta\;+\;\beta_{8}lnnrr\;+\;\beta_{9}lner\\ \;+\;\beta_{10}lnq\;+\;\mu \;+\;v\;+\;\varepsilon \end{array}$$

Except lnm is China's import volume, lnx is China's export volume, other variables are consistent with the trade flow model.

#### Results

##### Descriptive statistics

The results from the descriptive statistics analysis are shown in Table 1.

**Table 1:** Panel data description statistics.

**Variable**	***N***	**Average**	**S.D**.	**Min**	**Max**
Code	400	20.5	11.558	1	40
Year	400	4.5	2.876	0	9
lnq	400	−0.141	0.781	−2.02	3.35
lnofdi	400	6.733	4.773	−5.27	13.86
lndisp	400	1.657	0.451	−0.05	2.04
lndpcl	400	4.036	1.203	−2.98	6.67
lnlab	400	4.134	1.46	1.91	7.21
fta	400	0.055	0.228	0	1
lntf	400	4.14	2.055	−0.59	8.14
lngdp	400	2.36	1.699	−4.79	6.44
lnc	400	2.32	0.256	1.81	2.66
ln1edisp	400	2.727	0.403	1.28	3.08
lnm	400	5.078	2.522	−3.83	9.93
lnx	400	5.865	2.119	−2.2	9.63
lnnrr	400	0.248	2.156	−8.69	3.94
lner	400	−1.573	2.786	−5.82	5.44

##### Correlation analysis table

In this study, Pearson correlation analysis is used to ensure the effectiveness of each variable. If the correlation coefficient is lower than 0.3, it is a low correlation, 0.3–0.7 is a moderate correlation, and higher than 0.7 is a high correlation. From the analysis results, it can be found that there is a correlation between variables, and they are significant. The results show that the correlations between lntf and lnlab, lngdp and lnlab, lnm and lngdp, lnm and lnlab, and lnx and lnlab are above 0.7, which is a high correlation.

##### Unit roots test and Hausman test

Since the panel data is the same as the time-series data, the appearance of the unit root may cause the ols to have a false regression problem, which will affect the final empirical result. According to the unit root test conducted by Wang (2019) in the research on the trade effect of the B&R, this research will also test the unit root by Llc and ISP test (except dummy variables) before the regression. The results are shown that the prob of lndisp is over 0.1, whose data is uneven. Others are even. The analysis results show that lndisp has a unit root problem in both tests, lngdp and lntf have unit roots respectively, and the others remain stable at the 1% significant level. To confirm whether there is a stable causal relationship between the variables, the Westerlund cointegration test is performed on these variables to confirm whether there will be spurious regression in the results. Results the statistical index was 7.825. At the 1% significant level, the *p*-value was 0.00. It was rejected that there was no co-integration relationship between the variables, that is, there was a co-integration relationship, so there would be no pseudo-regression phenomenon. At the same time, the choice of the fixed-effect model and the random effect model of the three models are considered. The Hausman test was performed on the panel data used in this study to determine whether the model was a fixed-effects model or a random-effects model. It was found that the chi of the trade flow model was 39.8. The chi of the imported model is 77.36 and the chi of the exported model is 78.611, all of these are below the 1% significant level. Therefore, all three models should use individual fixed-effects models.

##### Regression analysis

After confirming the accuracy of the panel data, we performed individual fixed-effect regression analyses on the three models, and the results are shown in Table 2.

**Table 2:** Regression analysis results.

**Variable**	**lntf**	**lnm**	**lnx**
lngdp	0.02	−0.01	0.13
lnc	2.44***	1.46***	1.25***
lndisp/	−1.30	0.07	−0.14
lnofdi	0.02	0.02	0.01
lnq	0.09	0.04	0.20
lndpcl	−0.24***	−0.38***	−0.14
lnlab	−1.89*	−1.96	−2.40
fta	−0.15	−1.06**	0.26
lnnrr	0.04	0.06*	0.08**
lner	−0.01	−0.01	−0.03
constant	2.22	14.54**	15.93**

^***^p <0.01,

^**^p <0.05,

^*^p <0.1.

First of all, from the results of the trade flow model, we can see that foreign direct investment has an insignificant positive correlation of 0.02 on trade flow. Within the range of 1% significant level, the level of consumer ability has a negative correlation of 0.24 with trade flow China's economic level has a positive correlation of 2.44 with the flow among the 10 significant levels. Labor has a negative correlation of 1.89. Secondly, from the results of the trade import model, it can be seen that foreign direct investment has a positive relationship of 0.02 to imports, and within the range of the 1% significant level, China's GDP, consumer capacity differences, and imports have a positive correlation of 1.46 and a negative correlation of 0.38. The proportion of natural resource rent has a positive correlation of 0.08 at the 10% significance level. Finally, it can be seen from the results of the trade export model that foreign direct investment has an insignificant 0.01 proportional relationship to it. At the 1% level, China's GDP is proportional to 1.25. In the 5% significant case, differences in consumer ability and the proportion of natural resource rents have positive correlations of 0.19 and 0.07 for exports, respectively. The three trade models that can be finally obtained are:


$$\begin{array}{l} lntf_{ic}\;=\;0.02lnqdp\;+\;2.44lnc-1.30lndisp\;+\;0.02lnofdi\\ \;-0.09lnq-0.24lndpcl\;+\;1.89lnlab-0.15fta\\ \;+\;0.04lnrrr-0.01lner\;+\;\mu \;+\;v\;+\;\varepsilon \\ lnm\;=\;-\;0.01lnqdp\;+\;1.46lnc\;+\;0.02lnofdi\;+\;0.04lnq\\ \;-0.38lndpcl-1.96lnlab-1.06fta\;+\;0.06lnnrr\\ \;-0.01lner\;+\;0.07lnledisp\;+\;\mu \;+\;v\;+\;\varepsilon \\ lnx\;=\;0.13lnqdp\;+\;1.25lnc\;+\;0.01lnofdi\;+\;0.2lnq\\ \;-0.14lndpcl-2.40lnlab\;+\;0.26fta\\ \;+\;0.08lnnrr-0.03lner-0.14lnledisp\;+\;\mu \;+\;v\;+\;\varepsilon \end{array}$$

## Conclusion

### Discussion

This study analyzes trade flows, trade imports, and exports from 2010 to 2019. Concluded as follow:

(1) The increase in foreign direct investment can improve trade flows between China and countries along the B&R route. An in-depth analysis of import and export trade shows that foreign direct investment is more relevant to China's imports than its exports.
(2) The growth of the labor force population also has a negative correlation with imports and export. In the analysis of the current situation, it can be found that due to economic differences, there is still an imbalance in the development of the labor force in various regions.
(3) The expansion of China's economic scale can increase the scale of China's import and export trade among them, the consumption level gap between the two countries will restrict the degree of import and export trade.
(4) Although the impact is relatively small, the possession of natural resources can also promote the generation of trade, that is, the more mineral resources in the countries along the route, the larger the scale of import and export trade that can be carried out.

#### Strategies for foreign direct investment

This study finds that China's direct investment in countries along the B&R has a positive trade promotion effect, and can promote economic and trade development. Therefore, the establishment of foreign investment policies based on relevant laws, regulations, and foreign policies and smooth foreign investment can promote economic and trade development.

#### Strategies for labor force population

This study finds that the changes of labor force population in various countries hinder the trade flow between China and countries along the B&R. The increase of the labor force leads to serious employment and rising a unemployment rate. At present, China is constantly investing in manufacturing. The infrastructure construction and the employment opportunities of labor force in countries along the B&R should be improved.

#### Strategies for countries with different economic levels

The study found that when GDP expands, it can expand its import and export scale. The difference in the consumption capacity of trading countries is also a factor hindering the development of trade. However, the economic level of many countries along the route is stagnant, and the consumption level remains depressed. The joint communique of the round table in 2021 pointed out that the developing country was still struggling with poverty and economic growth. Especially since the COVID-19 pandemic, the countries along the route have been in constant conflict on people's livelihood issues, and the poverty reduction process has been impacted. Therefore, the mechanism and path of poverty reduction needed to be continued to explored. For example, poverty reduction knowledge and experience with countries along the route according to local conditions should be shared, and extensive cooperation with the Asian infrastructure investment bank to build a long-term and reasonable mechanism should be carried out.

#### Strategies for the impact of natural resources on import and export trade

The proportion of natural resource rent can represent the occupation of natural resources by the state. From the research and analysis, it is found that whether it is import trade or export trade, natural resources will have a significant impact on it. However, the background of the times will have different effects. When natural resources are abundant, but the poor ecological environment and the continuous expansion and urbanization of various countries still will not have reasonable development. This is why it is reasonable for Chinese enterprises to invest in supporting the green development of the B&R in route with environmental protection standards and practices, and to deepen energy cooperation and investment.

This study analyzes the data from 2010 to 2019, analyzes the trade gravity model based on China's total direct investment and trade flow, and puts forward the above strategies and suggestions.

### Originality/value

#### Comparison with past research

As for the research on the impact of direct investment on trade flows of countries along the B&R, the contribution of this research is to update the timeliness data in this field. The data used are from 2019, which is only analyzed to 2018 compared with the previous research. It is carried out with varying degrees of openness and exchange rate as variables. The model not only uses the trade gravity model to study the trade flow, but also uses the Tinbergen model to study the import and export trade. The research scope is broader. Most scholars focus on export trade, while this study focuses on import trade which was less studied. The research results and findings can make the follow-up researchers consider more factors in the research, and make the research results more objective, complete and comprehensive.

#### Difference comparison of models

Compared with different models used in previous studies, most of the previous relevant research literature used the stochastic frontier analysis model (SFA) (Li, 2016; Xu, 2017; Fang, 2018; Yang, 2020; Cao, 2022) or trade gravity model (Li et al., 2020; Shahriar et al., 2020) to analyze export trade, trade efficiency, the export potential and its influencing factors and the efficiency of direct investment. Only the optimal value of nonresistance trade is considered, and the result slightly deviates from the actual situation. This study uses the trade gravity model modified by Tinbergen, who won the Nobel Prize in 1969, and Tinbergen's trade gravity model for regression analysis, which is more suitable for the research of import and export trade.

### Practical implications

This study uses two trade gravity models to analyze import and export trade based on different factors and aspects. Therefore, the analysis results provide a factor analysis of the influence of enterprises and society in foreign direct investment (FDI). When specific factors affect the results of import and export trade, enterprises can conduct research and analysis on foreign direct investment (FDI) according to the status quo of important factors, especially the differences in labor force population, natural resources, consumption capacity, and the signing of trade agreements. When the analysis finds that specific factors affect the results of import and export trade, enterprises can formulate foreign direct investment (FDI) strategies based on the status quo of important factors, especially differences in labor force population, natural resources, consumption capacity, and trade agreement signed.

### Study limitations

Due to political crises and military conflicts in some countries along the B&R over the years, it is impossible to search for complete information. Therefore, this study only uses the commodity trade situation of 40 countries with complete information among the 64 countries along the B&R route, which is regional. While analyzing the model statically, the trade model has yet to take into account more complete dynamics such as immigration, net asset flows, and FDI flows. In terms of technology and data sources, it is impossible to enter high-authority databases to intercept data, such as the Heritage Foundation, Telecommunication Union databases, etc., which cannot be included in the database due to restrictions on network authority.

## Data availability statement

The original contributions presented in the study are included in the article/supplementary material, further inquiries can be directed to the corresponding author/s.

## Author contributions

All authors listed have made a substantial, direct, and intellectual contribution to the work and approved it for publication.

## Conflict of interest

The authors declare that the research was conducted in the absence of any commercial or financial relationships that could be construed as a potential conflict of interest.

## Publisher's note

All claims expressed in this article are solely those of the authors and do not necessarily represent those of their affiliated organizations, or those of the publisher, the editors and the reviewers. Any product that may be evaluated in this article, or claim that may be made by its manufacturer, is not guaranteed or endorsed by the publisher.
